# Neural correlates of integrated self and social processing

**DOI:** 10.1093/scan/nsaa121

**Published:** 2020-09-09

**Authors:** Laura Finlayson-Short, Christopher G Davey, Ben J Harrison

**Affiliations:** Melbourne Neuropsychiatry Centre, Department of Psychiatry, The University of Melbourne and Melbourne Health, Victoria 3052, Australia; Orygen, Melbourne, Victoria 3052, Australia; Centre for Youth Mental Health, The University of Melbourne, Melbourne, Victoria 3052, Australia; Melbourne Neuropsychiatry Centre, Department of Psychiatry, The University of Melbourne and Melbourne Health, Victoria 3052, Australia; Melbourne Neuropsychiatry Centre, Department of Psychiatry, The University of Melbourne and Melbourne Health, Victoria 3052, Australia

**Keywords:** self-referential processing, social processing, ventromedial prefrontal cortex, default mode network

## Abstract

Self-referential and social processing are often engaged concurrently in naturalistic judgements and elicit activity in overlapping brain regions. We have termed this integrated processing ‘self-other referential processing’ and developed a task to measure its neural correlates. Ninety-eight healthy young people aged 16–25 (M = 21.5 years old, 67% female) completed our novel functional magnetic resonance imaging task. The task had two conditions, an active self-other referential processing condition in which participants rated how much they related to emotional faces and a control condition. Rating relatedness required thinking about oneself (self-referential processing) and drawing a comparison to an imagined other (social processing). Self-other referential processing elicited activity in the default mode network and social cognition system; most notably in the ‘core self’ regions of the medial prefrontal cortex and posterior cingulate cortex. Relatedness and emotional valence directly modulated activity in these core self areas, while emotional valence additionally modulated medial prefrontal cortex activity. This shows the key role of the medial prefrontal cortex in constructing the ‘social-affective self’. This may help to unify disparate models of medial prefrontal cortex function, demonstrating its role in coordinating multiple processes—self-referential, social and affective processing—to allow the self to exist in a complex social world.

## Introduction

The neuroscientific study of the self has gained considerable momentum over the past decade, particularly in the context of human neuroimaging research. Much of this work has focused on understanding the neural basis of self-referential cognition or thinking about oneself. This is typically examined by experiments that require one to reflect on and make judgements about one’s own qualities and characteristics but can also involve recognizing one’s own face or voice. Despite the importance of these studies, there is growing recognition that the study of self-referential processes should extend to the social domain, whereby self-appraisals are made directly in relation to others (Denny *et al.*, 2012; Northoff, 2013; Herold *et al.*, 2016; Yamawaki *et al.*, 2017). Indeed, some have argued that self-referential and social processes are inextricably linked, because the self is inherently socially constructed (Forgas and Williams, 2002; Northoff, 2013). When we accumulate social information about ourselves, both good and bad, it is used to inform our sense of self (Mead, 2015). Similarly, the ability to hold a mental representation of ourselves allows us to model the same subjective experience in others and to exist effectively in a social system (Forgas and Williams, 2002; Uddin *et al.*, 2007; Molnar-Szakacs and Uddin, 2013). As such, self- and other-referential processes frequently overlap in real-life social situations. Understanding the integration of these two processes—‘self-other referential processing’—therefore promises to shed light on how the self is constructed in natural social settings.

Functional magnetic resonance imaging (fMRI) studies have shown that self-referential processing is associated with activation of cortical midline structures, including the medial prefrontal cortex (MPFC) and posterior cingulate cortex (PCC), as well as lateral posterior areas, such as the inferior parietal lobule and temporoparietal junction (TPJ) (Northoff and Bermpohl, 2004; Northoff *et al.*, 2006; van der Meer *et al.*, 2010; Qin and Northoff, 2011; Mak *et al.*, 2017). These regions are collectively recognized as the ‘default mode network’ (DMN), whose activity is characteristically elevated under passive resting-state conditions, when spontaneous self-referential thoughts dominate. Studies investigating other-referential processing also consistently implicate the DMN, along with other areas that commonly underlie social cognition (hereunder referred to as the social cognition system or SCS) and have previously been referred to as the extended mirror neuron system. These include the inferior frontal gyrus, anterior insula, parietal regions, ventral premotor and supplementary motor cortices (Uddin *et al.*, 2007; Molnar-Szakacs and Uddin, 2013). These two networks, although overlapping, appear to be associated with different forms of other-referential processing. The SCS appears to facilitate embodied simulation, which is an automatic and pre-cognitive process in which actions and emotions perceived in the physical domain are mapped onto our inner representations of actions and emotions (Molnar-Szakacs and Uddin, 2013). SCS activation is also seen in self-referential studies that involve sensory stimuli, like perceiving one’s own face or voice (Uddin *et al.*, 2007; Molnar-Szakacs and Uddin, 2013; Hu *et al.*, 2016). By contrast, the DMN is linked to the higher-level cognitive abstraction of one’s own and others’ actions and emotions, or ‘mentalizing’ (Uddin *et al.*, 2007; Molnar-Szakacs and Uddin, 2013; Hu *et al.*, 2016).

Currently, very few studies have examined the integration of self- and other-referential processes, especially regarding how they might be engaged in natural social settings. One early study examined both self- and other-referential processing using a task that was likely to have evoked both embodied and mentalizing levels of processing (Ochsner *et al.*, 2004). They found common engagement of DMN regions, including the MPFC, as well as key nodes of the SCS. Self-referential judgements were more specifically engaging of MPFC subregions compared to other-referential judgements. In the current study, we sought to extend this work by investigating the neural basis of integrative self-other referential processing using a novel social judgement task. Specifically, we had participants make self-other referential judgements about how much they would relate to a person based on physical appearance and imagined personality. This paradigm extends previous self-referential processing paradigms, in which participants judge whether a trait adjective describes them or whether objects are self-relevant. This task involves more complex self and social processing than existing paradigms and we believe maintains a similarly high level of ecological validity, as the type of judgement employed is something that people engage in on a daily basis. Additionally, the results will provide novel evidence of the neural correlates of increasing self-other relatedness; separating the affective value of relating to another person from the basic processes of self and social cognition. To this end, our primary aim was to investigate the extent to which simple appraisals of self-other relatedness would modulate key areas of the DMN and SCS. Additionally, we sought to understand the extent to which such appraisals may be influenced by the affective content of others’ facial expressions. We included more than one emotional expression in an attempt to increase the ecological validity of the task, because when people meet strangers, they generally portray a medium level of emotional expressiveness (Hess *et al.*, 1995). We hypothesized that self-other referential judgements would be broadly activating of DMN and SCS regions, but that participants’ appraisals of self-other relatedness would be most directly associated with MPFC activity, consistent with its higher-level role in self-referential cognition (Davey *et al.*, 2016). Furthermore, we anticipated that this effect would be most apparent during the self-other appraisal of positive affect, given broader evidence that the MPFC preferentially responds to self-relevant stimuli with perceived positive affective value (Roy *et al.*, 2012; Harrison *et al.*, 2017).

## Methods

### Participants

Healthy young people (*N* = 127) between the ages of 16 and 25 gave their informed consent to participate in the study and completed the full study protocol. The study was approved by the University of Melbourne Human Research Ethics Committee and was carried out in accordance with the Declaration of Helsinki. Participants were recruited to the study via online classified advertisements, electronic student noticeboards and by word of mouth. Participants were eligible if they: (i) spoke English competently (assessed subjectively in a phone interview), (ii) had no current or past diagnosis of a mental illness based on participant history and the Structured Clinical Interview for DSM-5 Disorders, Research Version (SCID-5-RV) (First *et al.*, 2015), (iii) were not being treated with psychoactive medications, including antipsychotic, antidepressant, mood-stabilizing and sedative-hypnotic medications, (iv) were not dependent on substances and/or alcohol, determined by the World Health Organization Alcohol, Smoking and Substance Involvement Screening Test Version 3.0 (WHO ASSIST Working Group, 2002), (v) had no major brain abnormalities as indicated by MRI and (vi) had no further contraindications to MRI.

Twenty-nine participants were subsequently excluded; 21 due to excessive head motion during fMRI, 1 due to a technical failure during fMRI acquisition, 2 for incidental findings and 5 for meeting criteria for a clinical diagnosis on the SCID-5-RV. A total of 98 participants (67.35% female; M = 21.49 years old, SD = 2.20 years) were included in further analyses. The sample size was determined based on our past studies of this nature (see: Davey *et al.*, 2016). All participants completed the Self-Concept and Identity Measure (SCIM) (Kaufman *et al.*, 2014), which measures disturbed identity (discontinuity in values and beliefs, and overreliance on others for defining identity), consolidated identity (knowing who one is, consistency in values and beliefs, self-worth) and a lack of identity (feelings of emptiness and not knowing who one is). It has a total score of 189, with higher scores representing greater levels of identity disturbance. The SCIM was intended to measure the full spectrum of identity, from healthy to clinically disturbed. It was validated in two linked studies, which revealed and confirmed the three-factor structure described earlier (Kaufman *et al.*, 2014). The measure had high internal consistency, test–retest reliability and construct validity. It also correlated with measures of psychopathology, particularly those characterized by identity disturbance like borderline personality disorder and major depressive disorder.

### fMRI task

**Fig. 1.**
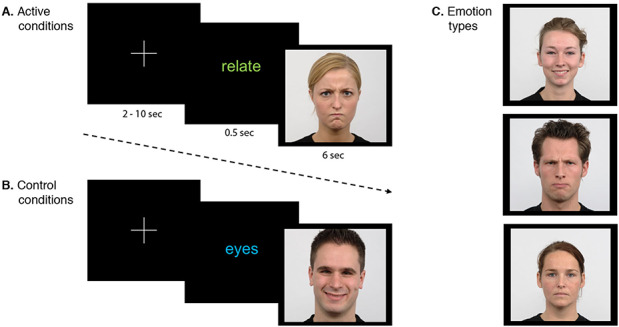
Experimental paradigm. Fixation cross followed by a question prompt and an emotional face stimulus in the active (relate) (A) and control (eyes) (B) conditions. Examples of the three emotion types available in both conditions are in (C); happy, angry and neutral.

We developed a novel ‘social judgement task’ that sought to evoke integrated self-other referential appraisals by having participants rate how much they felt they would relate to a person based on a picture of their face, featuring one of three facial expressions (Figure 1). During a pre-scan training session, participants were instructed that they would be presented with a series of people’s faces and be asked two possible questions about them: ‘how much do you relate to this person?’ and ‘how far apart are this person’s eyes?’. To answer the first question, they were instructed to think about the potential qualities and characteristics of the person in relation to their own, and whether they would be similar or relatable. Participants responded to the question using a button box on a three-point scale, indicating (i) that they did not relate to that person at all, (ii) that they related to them somewhat or (iii) that they related to the person very much. For control conditions, participants judged how far apart people’s eyes were based on subjective judgements. They could answer that the eyes were (i) very close together, (ii) somewhat close together or (iii) far apart. Participants were told that they must answer each question while the photo remained on the screen but should take their time to respond within that window.

The task comprised six conditions (three active and three control), all including an instruction followed by a face stimulus, which either displayed an angry, happy or neutral expression. The six conditions were therefore: ‘happy relate’ (relate question with a happy face), ‘angry relate’ (relate question with an angry face), ‘neutral relate’ (relate question with a neutral face), ‘happy eyes’ (eyes question with a happy face), ‘angry eyes’ (eyes question with an angry face) and ‘neutral eyes’ (eyes question with a neutral face).

In total, the task comprised 72 events. The instruction period interval for each event was 500 ms and involved the presentation of the word ‘relate’ (representing the relate question) or ‘eyes’ (representing the eyes question), rendered in blue or green lowercase text, respectively, on a black background. The instruction was followed by an emotional face stimulus with an interval of 6000 ms, with an interval of 2000 to 10000ms before the presentation of the next face. All faces were taken from the Radboud Faces Database (Langner *et al.*, 2010). There were 18 female and 18 male faces included in the task, comprising a total of 36 faces. Twelve faces (six male and six female) were assigned to each of the three emotion types, with each set of faces being displayed twice; once in a relate condition and once in an eyes condition. Emotional faces were pseudo-randomized, with an established order of presentation of each condition, but with randomization of the particular face shown. Event timing and sequencing was optimized using the Mac OSX version of optseq2 (https://surfer.nmr.mgh.harvard.edu/optseq/). Three versions of the task were generated to counterbalance the set of faces displayed for each emotion type. It was presented using Paradigm software (http://www.paradigmexperiments.com) on a Dell computer via MRI-compatible high-resolution goggles (VisuaStim Digital System, Resonance Technology Inc., Northridge, CA). Participants’ responses were registered with a fORP curved 4-button response box (Cambridge Research Systems Ltd).

### Image acquisition

A 3T General Electric Discovery MR750 system equipped with an eight-channel phased-array head coil was used in combination with ASSET parallel imaging. The functional sequence consisted of a single-shot gradient-recalled EPI sequence in the steady state (repetition time, 2 s; echo time, 35 ms; and pulse angle, 90°) in a 23-cm field-of-view, with a 64 × 64-pixel matrix and a slice thickness of 3.5 mm (no gap). Thirty-six interleaved slices were acquired parallel to the anterior–posterior commissure line with a 20° anterior tilt to better cover ventral prefrontal cortical brain regions. The total sequence time was 12 min 12 s, corresponding to 362 whole-brain echo-planar imaging volumes. To assist with noise reduction and head immobility, all participants were fitted with insert-ear protection and their heads were supported with foam-padding inserts.

### Image pre-processing

Imaging data was transferred to a Unix-based platform that ran MATLAB Version 9.3 (The MathWorks Inc., Natick, USA) and Statistical Parametric Mapping Version 12 (Wellcome Trust Centre for Neuroimaging, UK). Motion correction was performed by realigning each participant’s time series to the first image using least-squares minimization and a six-parameter rigid body transformation. Motion fingerprint (Statistical Parametric Mapping toolbox) (Wilke, 2012) was used to quantify participant head motion. Participants were excluded if movement exceeded 2 mm mean total displacement or 2.5 mm maximum scan-to-scan displacement. After slice-timing correction, the realigned functional images were then spatially normalized to the International Consortium for Brain Mapping template, resliced to 2 mm isotropic resolution and smoothed with a 6 mm full-width-at-half-maximum Gaussian filter.

### Subject-level fMRI analysis

Three subject-level general linear models were constructed. The first (‘Model 1’) separated the events into the six conditions described earlier. The second (‘Model 2’) grouped events according to participants’ ‘relatedness’ responses (‘not at all’, ‘somewhat’ or ‘very much). The third (‘Model 3’) grouped events according to participants’ ‘eye distance’ responses (‘close together’, ‘somewhat close’ ‘far apart’). With ‘Model 1’, we intended to broadly examine brain responses to the task, as well as their modulation by the affective content of facial expressions. ‘Model 2’ was intended to examine the degree to which brain activity was directly modulated by participants’ ratings of self-relatedness. ‘Model 2’ was estimated in 90 participants (65.56% female; M = 21.38 years old, SD = 2.14 years), as 8 participants did not provide adequate responses across all conditions (did not respond with all 3 response types). ‘Model 3’ was estimated in 91 participants (68.10% female; M = 21.37 years old, SD = 2.13 years), as 7 participants did not provide adequate responses across all conditions (did not respond with all 3 response types).

Primary regressors specifying the onset and duration of each event for both models were entered for each participant. The events in the models were convolved with a canonical hemodynamic response function. The rest-fixation epochs served as the implicit baseline. A high-pass filter (1/128 s) accounted for low-frequency noise, while temporal autocorrelations were estimated using a first-order autoregressive (AR1) model. Regression coefficient estimates (betas) were calculated using a Restricted Maximum Likelihood approach and primary contrast images were estimated for each participant: all relate > rest and relate > eyes contrasts (‘Model 1’); self-relatedness: relate very much > relate not at all, relate very much > relate somewhat and relate somewhat > relate not at all contrasts (‘Model 2’) and eye distance: eyes far apart > eyes close together, eyes far apart > eyes somewhat close together, eyes somewhat close together > eyes close together (‘Model 3’).

### Group-level fMRI analysis

Group-level (GLM) analyses were performed using the summary statistics approach to random-effects analyses. Subject-level contrast images for ‘Model 1’ were carried forward to the second level, where a full factorial model was estimated. This full factorial modelled two factors: condition and emotion. Main effects and interactions were examined for this model, with post-hoc pairwise comparisons estimated where significant effects were found. Simple main effects and post-hoc pairwise comparisons were masked by the resulting map of the relevant main effect or interaction and thresholded at small-volume corrected (SVC) false discovery rate (FDR) (*P *< 0.05, K_E_ = 10 voxels), with an entry threshold of *P *< 0.001 uncorrected. The main effect of emotion and interaction of condition and emotion were masked by the resulting simple main effect of condition (relate > eyes) and thresholded at SVC-FDR (*P *< 0.05, K_E_ = 10 voxels), with an entry threshold of *P *< 0.001 uncorrected. For ‘Model 2’, one-sample *t*-tests were estimated for the three self-relatedness subject-level contrasts, while for ‘Model 3’, one sample *t*-tests were estimated for the three eye distance subject-level contrasts. These models were examined by applying a whole-brain FDR threshold of *P*_FDR_* *< 0.05 and a minimum cluster extent (K_E_) of at least 10 contiguous voxels.

### Task performance associations

We examined associations between behavioural and brain measures of task performance with trait identity (total SCIM score) and age. Associations between task performance (relatedness ratings), trait and demographic measures were estimated by Pearson’s correlation in SPSS Statistics for Macintosh Version 24 (IBM Corp, USA). Missed responses were imputed. Imputed values were based on the mode of responses for the question across all participants. Associations between brain activity, SCIM and age were estimated by specifying them as covariates within one-sample *t*-tests of the very much > not at all (‘Model 2’) contrast, while gender differences were estimated via a two-sample *t*-test analysis of the very much > not at all contrast. These analyses were restricted to an inclusive mask of the self-relatedness very much > not at all group result, thresholded at SVC-FDR *P *< 0.05, K_E_ = 10 voxels (entry threshold of *P *< 0.001 uncorrected).

## Results

### Behavioural task performance

A repeated-measures ANOVA of reaction time (Supplementary Table 1) showed a significant interaction between condition and emotion type. Bonferroni-corrected post-hoc tests of simple main effects (Supplementary Table 2) confirmed that participants took significantly longer to react to neutral faces in the relate conditions compared to the eyes conditions. Participants also took longer to react to neutral faces than happy faces within the relate conditions. In the eyes conditions, participants took longer to react to happy and angry faces compared to neutral faces. A repeated-measures ANOVA of participant relatedness ratings indicated that there was a significant main effect of emotion type. Post-hoc pairwise comparisons (Bonferroni corrected) confirmed that ratings of relatedness were significantly different between all emotion types, with participants relating the most to happy faces, followed by neutral faces, then angry faces. The mean difference in relatedness scores was most apparent between happy and angry faces, indicating a clear effect of valence on appraisals of self-relatedness. Participants’ mean SCIM score was relatively low, given a total possible score of 189 (M = 66.12, SD = 19.78).

### Functional MRI

#### Model 1: self-other referential judgement.

**Fig. 2.**
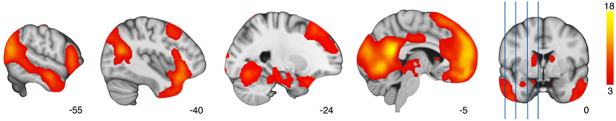
Activation map representing self-other referential processing (simple main effect of condition: relate > eyes). Small volume correction applied, main effect of condition thresholded at *P*_FDR_ < 0.05, K_E_ = 10, entry threshold of *P*_Uncorrected_ < 0.001, K_E_ = 10. Left = left.

There was a significant main effect of condition across much of the brain (Supplementary Table 3). The simple main effect of condition showed which of this activity was associated with the relate > eyes contrast (Figure 2 and Supplementary Table 4). This showed widespread significant activation of brain regions routinely implicated in self- and other-referential processing, as well as general emotional-affective responding. This pattern of activation included the extended MPFC, spanning its ventral and dorsal divisions, and extending to the rostral anterior cingulate cortex. Extensive activation of the posterior medial wall cortex was also observed, spanning the posterior cingulate and retrosplenial cortex, and the cuneus-precuneus. Other prominently activated areas included the middle temporal gyrus/TPJ, inferior parietal lobule, hippocampus-amygdala, frontal operculum-inferior frontal gyrus, striatum, ventral diencephalon and medial thalamus.

**Fig. 3.**
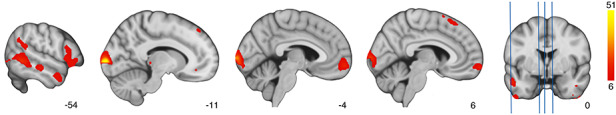
Activation map representing emotional processing (main effect of emotion). Small volume correction applied, relate > eyes, itself small volume corrected as in Figure 2, entry threshold of *P*_Uncorrected_ < 0.001, K_E_ = 10. Left = left.

There was a significant main effect of emotion in the ventral and dorsal MPFC, including the gyrus rectus and frontal pole; the anterior cingulate cortex; the precuneus; the temporal cortex extending to the angular gyrus; lateral frontal areas, including the inferior frontal gyrus extending to the lateral and posterior orbital gyri; the postcentral gyrus and supplementary motor cortex; the thalamus and visual areas (Figure 3 and Supplementary Table 5).

There was no significant interaction between condition and emotion when masked by the simple main effect of condition (relate > eyes). This indicates that there were no areas of increased activity related to the processing of emotional faces in the relate compared with the eyes conditions. Rather, differential activation seen in the main effect of emotion contrast was due to basic differences in emotional face processing across all relate and eyes conditions. As such, no further post-hoc tests were carried out.

#### Model 2: modulation by degree of self-relatedness.

**Fig. 4.**
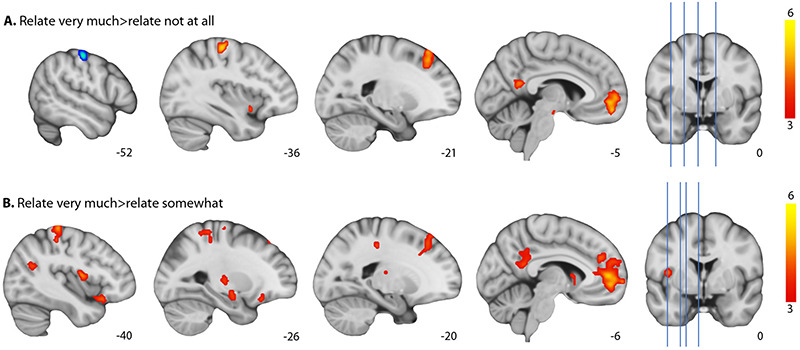
Activation map representing increasing relatedness: relate very much > relate not at all (A) and relate very much > relate somewhat (B), with activation in orange and deactivation in blue. Thresholded at *P*_FDR_ < 0.05. Left = left.

Participants’ ratings of self-relatedness (very much > not at all responses) were observed to directly modulate several areas that were active in response to overall task performance (‘Model 1’), including most notably the ventral MPFC, PCC and ventral precuneus. Additional areas that were modulated by the self-relatedness ratings included the left superior frontal gyrus; ventral anterior insula; left pre- and post-central gyri; dorsal midbrain (∼substantia nigra, ventral tegmentum) and visual areas. There was also significant deactivation associated with increasing relatedness in the primary somatosensory cortex (Figure 4A and Supplementary Table 6).

The very much > somewhat contrast analysis identified similar activation of core DMN areas including the ventral and dorsal MPFC, anterior and posterior cingulate cortices, and ventral precuneus. There was also activation in the left superior frontal gyrus, left pre- and post-central gyri, superior parietal lobule, angular gyrus, supramarginal gyrus, left anterior insula, temporal areas, inferior frontal gyrus, striatum (putamen, ventral caudate), thalamus and amygdala (Figure 4B and Supplementary Table 6). The somewhat > not at all contrast was not significant.

#### Model 3: modulation by eye distance.

Participants’ ratings of eye distance (far apart > close together) were associated with deactivation in a small cluster covering the right middle and superior frontal gyri (Supplementary Table 7). No other eye distance contrasts reached significance.

### Brain and behavioural associations

Significant associations were identified between total SCIM score and task performance (relatedness ratings) in the happy relate (*r *= −0.41, *P *< 0.0001) and angry relate (r = 0.32, *P *= 0.001) conditions. These results suggest that participants with a less stable self-concept related less to happy faces and more to angry faces. We also identified a significant positive correlation between activation in the left superior frontal gyrus in the very much > not at all contrast and SCIM scores (Supplementary Figure 1).

## Discussion

In this study, we have defined self-other referential processing as the integrative appraisal of self in relation to others. We examined it by having participants appraise the degree to which they would relate to a person based on physical appearance and imagined personality. Such processes are likely to be frequently engaged in real-life social situations and may be important for understanding how the self is constructed in the context of social relationships.

Overall performance of the task was associated with the activation of widely distributed brain regions throughout the DMN and SCS, along with additional areas related to face processing, reward and emotion. This is due to the highly complex nature of the phenomenon under study. Self-other referential processing is likely to have engaged multiple component processes, ranging from basic face perception and emotion recognition to self- and other-referential processing, comparing the self to the other and making a higher-order value judgement. This complex processing cascade would necessarily engage a wide range of brain networks in concert. However, our main aim was to better understand the role of the DMN and its direct links to self-appraisal, along with the moderating roles of increasing relatedness and affect.

Our primary hypothesis was that self-other referential judgements would broadly activate DMN and SCS regions, with central involvement of the MPFC. Our results support this: self-other referential processing, across all affect types, was associated with robust activation of the major components of these networks, including prominent involvement of both the ventral and dorsal MPFC. Other key DMN regions were also involved, including the anterior and posterior cingulate cortices and medial temporal areas. SCS areas that were engaged included the inferior frontal gyrus, anterior insula, primary sensory and motor cortices, and the TPJ. This accords with the complex nature of our task, which should have engaged both low-level embodied and higher-level mentalizing aspects of self- and other-referential processing. In other words, in our task, participants should have considered not only the physical aspects of others but also their imagined thoughts, ideas and values. Previous work has suggested that embodied processing and mentalizing engage both the DMN and SCS during separate self- and other-referential conditions (Ochsner *et al.*, 2004; Trapp *et al.*, 2014). Our work extends these findings, demonstrating that these networks also co-activate during combined self-other referential processing.

Our findings align with a previous meta-analysis investigating self- and other-referential processing. van der Meer *et al.* (2010) proposed a combined self and other-referential processing system involving the key components of the DMN. They theorized that the anterior cingulate cortex allows attention to be directed toward the self and the ventral MPFC then tags incoming information as self-relevant. Meanwhile, the insula and PCC provide information regarding the internal bodily state and autobiographical memory, respectively, that could be relevant to either self- or other-referential processing. Finally, the dorsal MPFC mediates the decision-making process (i.e. ‘do I relate to this person?’). Our task should require a similar process and our findings showed significance for all of the regions included in van der Meer *et al.*’s (2010) model, in both the relate > eyes and self-relatedness results.

An additional meta-analysis of ventral MPFC subsystems by Roy *et al.* (2012) is also highly relevant to our findings. They found that two subsystems of the ventral MPFC were engaged during both self-referential and social processing. First, an ‘affect generation’ subsystem, consisting of the ventro-caudal ventral MPFC and subcortical areas associated with affect and reward processing. The second is the ‘simulation’ subsystem, including the anterior and dorsal ventral MPFC and PCC, which simulates internal models of the world. They are hypothesized to work together to support the generation of ‘affective meaning’, whereby the meaning of events is inferred from situational cues and context-appropriate behaviour is produced. Our data are consistent with the engagement of both proposed subsystems and consistent with the Roy model. If it is engaged in correctly, our task should involve affective meaning generation: taking in visual information, using this to mentally simulate self and other, then generating appropriate affect and decision-making.

Importantly, individuals’ appraisals of self-other relatedness were confirmed to directly modulate ventral MPFC activity, together with the PCC and midbrain-striatal areas. The results of our analysis of eye distance ratings confirm that activity in these areas was not solely related to more strongly endorsing a trait, but rather is unique to self-relatedness. The involvement of the ventral MPFC and PCC also supports the previous theory of our team (Davey *et al.*, 2016) and others (Buckner *et al.*, 2008; Andrews-Hanna *et al.*, 2010) that these regions comprise the core hubs of the DMN. Our results suggest that these previous self-referential and resting state findings generalize into the social domain. The ‘core self’ areas also appear to track the degree to which appraisals were of a positive-affective nature, with greater activity in these regions when individuals related more to others. Surprisingly, these results were not mirrored in the emotional valence analysis, despite the behavioural finding that participants related most to happy faces and that scores of relatedness to happy faces correlated negatively with poor self-concept. Rather, there was increased ventral MPFC activity across all conditions, dovetailing with previous work that found that the ventral MPFC tracks an individual’s current subjective experience of emotion (Winecoff *et al.*, 2013). We postulate that a positive self-concept likely leads people to relate more to happy faces, but this relationship may not be mediated by ventral MPFC activity, despite the ventral MPFC tracking positive emotional valence more broadly.

We further observed that participants with poorer self-functioning showed greater activation of the left superior frontal gyrus when relating to others. While not recognized as part of the DMN, the left superior frontal gyrus frequently coactivates with the network (van der Meer *et al.*, 2010). The region has strong functional connectivity with the DMN, as well as the cognitive control network (CCN), and may act as a connecting node between the two networks (Li *et al.*, 2013). Switching between the DMN and CCN is necessary when tasks require both internally (DMN) and externally (CCN) directed attention, as active self- or other-referential tasks do. As such, our finding could suggest that poor self-functioning is related to a dysfunction in this network linkage. However, this interpretation is speculative and should be tested directly by future research.

This study had some limitations. Our task attempted to probe simple self-other judgements. To ensure ecological validity, the instruction in the relate condition was open-ended and so allowed a degree of interpretation by participants. It is possible that, despite our instructions, some participants made shallow judgements based on similarity of physical attributes between self and other. They may also have made judgements based on how much they were motivated to approach the depicted person, which might have included confounding factors such as age, racial bias and sexual attractiveness. We used stimuli that exclusively pictured Caucasian young people with a sample made up of a majority of Caucasian young people. We did not control for attractiveness, which may have affected the results. Varying our stimuli based on race, age and attractiveness, and investigating the effects of these factors on our findings could have allowed us to determine whether these factors confounded the results. We also did not collect information on the kinds of heuristics participants used to make integrated self-other referential judgements, which would have shed light on the factors influencing their decision making. The idea that participants were not overly relying on the use of heuristics to make these judgements was partly validated by associations between performance and self-functioning scores, as well the modulation of relatedness ratings by affective valence. However, the modulation of relatedness ratings by valence could also reflect self-serving biases towards relating to positive emotions and does not mean that other biases could not have influenced decision making.

We also combined both self- and other-referential processing, and embodied processing and mentalizing in our task. Including embodied processing via explicitly presenting participants with a photo of another person ensured that they engaged in other-referential processing. However, it would be worthwhile to investigate whether combined self- and other-referential processing that uses embodied processing or mentalizing alone, rather than both, produces consistent or divergent results. Adding separate self- and other-referential processing conditions would have allowed us to disentangle the separate effects of thinking about the self and others. Comparing these separated conditions to integrated self-other referential processing would have revealed the degree to which the neural correlates of integrated judgements overlapped with or were distinct from those of separate self- and other-referential processing. Future research should consider this question. Nevertheless, our task has been the first to target integrated self-other referential processing in healthy young people and outline its neural correlates, and it is novel in that respect.

Including additional control conditions could also have improved the task. The eyes conditions controlled for multiple factors at once, including making a judgement about another person, using a three-point rating scale, face perception and button pressing. This model was chosen to simplify the task and reduce the time spent in the scanner by participants. However, adding a condition that required participants to judge whether another person’s eyes were closer together or further apart than one’s own could have isolated the difference between making internal and external feature assessments during self-other referential processing.

As mentioned earlier, it is also possible that racial biases influenced participants’ responses to the Caucasian faces included in the task. We did not collect information on implicit or explicit racial biases, so cannot be sure of their possible effect on our results. Future studies should consider the influence of such biases. Additionally, some participants could have interpreted the faces in the control condition as self-relevant and engaged in implicit self-referential processing, similar to previous research (Rameson *et al.*, 2010; Yang *et al.*, 2014). However, given the extent to which areas typically associated with self-reference (i.e. the DMN) were recruited in the contrast relate > eyes, this suggests that if any self-reference did occur in the control condition it was likely very minor. Previous work also observed this implicit self-reference effect when participants viewed stimuli with personally relevant context and were then asked non-self-related questions about them. In contrast, our task presented participants with images of strangers with no further contextual information, amounting to stimuli low in self-relevant cues compared with those of Rameson *et al.* (2010) and Yang *et al.* (2014). Finally, we used a self-report measure of self-functioning to ensure that our neuroimaging results were related to real-world behavioural differences. Using an additional informant-report measure of self-functioning would have further strengthened the study but was outside the scope of the project.

Notwithstanding these limitations, this study has been the first to show that the neural correlates of self-other referential processing appear to rely principally on the DMN and SCS, with a key integrative role for the MPFC as part of the core DMN. Our findings suggest that, while integrated self-other referential processing likely engages multiple cognitive operations that rely on distributed areas of the DMN and SCS, pro-social self-judgements (appraising oneself as more related to some than others) appear to rely on the functional operation of the core DMN areas. This is a finding that may help to unify disparate models of MPFC function, which emphasize its role in affect, social cognition and self-functioning (Roy *et al.*, 2012; Delgado *et al.*, 2016; Hiser and Koenigs, 2018). The need for a ‘social-affective self’, in other words, for the ability to engage in self-referential processing that is inextricably linked to its social context and affective in- and outputs, may explain why the MPFC appears to have multiple distinct functions. Rather than acting discretely to produce separate processes, the MPFC’s role may be to integrate these processes to allow the self to exist in a complex social world.

## Supplementary Material

nsaa121_Supp
