# Supplementary material for: Neural correlates of integrated self and social processing
Source: Soc Cogn Affect Neurosci. 2020 Sep 9;15(9):941–9. doi: 10.1093/scan/nsaa121 (PMC7647375; doi:10.1093/scan/nsaa121)
Supplement: nsaa121_Supp [file nsaa121_supp.zip › nsaa121_scan-20-086-File006.docx]

**Neural correlates of integrated self and social processing**

**Supplementary Material**

*Supplementary Table 1.* Effects of condition and emotion on reaction time and relatedness ratings.

| **Variable** | **Post-Hoc Test** | **Active (Mean ± SD)** | | | **Control** | | | ***P*-Value** | | |
| --- | --- | --- | --- | --- | --- | --- | --- | --- | --- | --- |
|  |  | **Happy** | **Angry** | **Neutral** | **Happy** | **Angry** | **Neutral** | **Condition** | **Emotion** | **Condition x Emotion** |
| **Reaction Time** |  | 2229.61 ± 637.02 | 2294.35 ± 650.02 | 2375.54 ± 572.60 | 2293.37 ± 568.39 | 2355.68 ± 551.47 | 2213.620 ± 599.69 | .744 | .172 | <.001* |
|  | Simple Main Effect: Happy Faces | ✓ |  |  | ✓ |  |  | .235 |  |  |
|  | Simple Main Effect: Angry Faces |  | ✓ |  |  | ✓ |  | .259 |  |  |
|  | Simple Main Effect: Neutral Faces |  |  | ✓ |  |  | ✓ | <.001* |  |  |
|  | Simple Main Effect: Active Condition | ✓ | ✓ | ✓ |  |  |  |  | .011* |  |
|  | Simple Main Effect: Control Condition |  |  |  | ✓ | ✓ | ✓ |  | .005* |  |
| **Relatedness Ratings** |  | 2.31 ± .48 | 1.39 ± .38 | 1.71 ± .35 |  |  |  |  | <.0001* |  |

*Supplementary Table 2.* Pairwise comparisons arising from the repeated measures ANOVAs detailed in Table 1.

| **Variable** | **Post-Hoc Test** | **Pairwise Comparison** | **Mean Difference** | **Standard Error** | ***P*-Value** | **95% CI Lower Bound** | **95% Upper Bound** |
| --- | --- | --- | --- | --- | --- | --- | --- |
| **Reaction Time** | Simple Main Effect: Happy Faces | Happy Active – Happy Control | -63.76 | 53.30 | .235 | -169.564 | 42.043 |
|  | Simple Main Effect: Angry Faces | Angry Active – Angry Control | -61.33 | 54.05 | .259 | -168.62 | 45.96 |
|  | Simple Main Effect: Neutral Faces | Neutral Active – Neutral Control | 161.91 | 45.87 | .001* | 70.86 | 252.97 |
|  | Simple Main Effect: Active Condition | Happy Active – Angry Active | -64.72 | 47.83 | .54 | -181.29 | 51.81 |
|  |  | Happy Active – Neutral Active | -145.92 | 46.44 | .007* | -259.08 | -32.77 |
|  |  | Neutral Active – Angry Active | 81.19 | 49.68 | .317 | -39.87 | 202.25 |
|  | Simple Main Effect: Control Condition | Happy Control – Angry Control | -62.31 | 45.34 | .518 | -172.77 | 48.16 |
|  |  | Happy Control – Neutral Control | 79.75 | 34.73 | .072 | -4.88 | 164.38 |
|  |  | Neutral Control – Angry Control | -142.06 | 45.56 | .007* | -253.06 | -31.05 |
| **Relatedness** | Simple Main Effect: Active Condition | Happy Active – Angry Active | .92 | .07 | <.0001* | .74 | 1.09 |
|  |  | Happy Active – Neutral Active | .60 | .06 | <.0001* | .46 | .75 |
|  |  | Neutral Active – Angry Active | .31 | .05 | <.0001* | .20 | .42 |

All pairwise comparisons were Bonferroni corrected for multiple comparisons.

*Supplementary Table 3.* Significant activation associated with the main effect of condition. Activation thresholded at *P*_FDR_<.05, K_E_=10.

| **Anatomic Region** | **Cluster Size (mm^3^)** | **Peak MNI Coordinates** | | | ***Z*** |
| --- | --- | --- | --- | --- | --- |
|  |  | **x** | **y** | **z** |  |
| *Precuneus | 97626 | -4 | -54 | 20 | Inf |
| *Inferior temporal gyrus | 1806 | -52 | -60 | -14 | Inf |
| *Middle frontal gyrus | 1623 | 26 | 2 | 54 | Inf |
| *Precentral gyrus | 997 | 50 | 6 | 24 | Inf |
| *Precentral gyrus | 1020 | -46 | 2 | 26 | Inf |
| *Middle frontal gyrus | 1152 | -24 | 0 | 52 | Inf |
| *Inferior temporal gyrus | 2102 | 52 | -56 | -18 | Inf |
| *Middle frontal gyrus | 1066 | 44 | 42 | 28 | 5.57 |
| Fusiform gyrus | 102 | -32 | -46 | -16 | 5.45 |
| Middle frontal gyrus | 1300 | -46 | 50 | 12 | 5.01 |
| Cerebellum | 176 | -32 | -36 | -46 | 4.49 |
| Anterior orbital gyrus | 53 | -28 | 48 | -22 | 4.11 |
| Anterior insula | 81 | 36 | -2 | 4 | 4.03 |
| Anterior insula | 101 | 30 | 22 | -2 | 4.03 |
| Postcentral gyrus | 289 | -52 | -20 | 56 | 3.74 |
| Superior parietal lobule | 152 | -22 | -62 | 72 | 3.59 |
| Anterior insula | 50 | -38 | -4 | 4 | 3.49 |
| Anterior insula | 42 | -30 | 18 | 2 | 3.36 |
| Brain stem | 31 | 14 | -24 | -32 | 3.25 |
| Anterior orbital gyrus | 42 | 30 | 56 | -20 | 3.23 |
| Parietal operculum | 123 | -34 | -26 | 16 | 2.69 |
| Precuneus | 23 | 8 | -40 | 44 | 2.63 |
| Supplementary motor cortex | 16 | 6 | 22 | 46 | 2.55 |
| Central operculum | 25 | -66 | -12 | 10 | 2.48 |
| Postcentral gyrus | 10 | -40 | -34 | 70 | 2.32 |
| Postcentral gyrus | 13 | 26 | -34 | 64 | 2.31 |

*Clusters that also survive whole-brain FWE correction for multiple comparisons

*Supplementary Table 4.* Significant activation associated with self-other referential processing (simple main effect of condition: relate>eyes contrast). Small volume correction applied, main effect of condition thresholded at *P*_FDR_<.05, K_E_=10, entry threshold of *P*_Uncorrected_<.001, K_E_=10.

| **Anatomic Region** | **Cluster Size (mm^3^)** | **Peak MNI Coordinates** | | | ***Z*** |
| --- | --- | --- | --- | --- | --- |
|  |  | **x** | **y** | **z** |  |
| Precuneus | 31776 | -4 | -54 | 20 | Inf |
| vMPFC/dMPFC | 20431 | 4 | 58 | 10 | Inf |
| Middle temporal gyrus | 10581 | 58 | -62 | 12 | Inf |
| Postcentral gyrus | 92 | -52 | -20 | 56 | 3.91 |
| Transverse temporal gyrus | 90 | 40 | -26 | 12 | 3.67 |
| Putamen | 13 | -16 | 4 | -8 | 3.64 |
| Parahippocampal gyrus | 22 | 20 | -28 | -10 | 3.63 |

*Supplementary Table 5.* Significant activation associated with the main effect of emotion. Small volume correction applied, relate>eyes, itself small volume corrected as in Supplementary Table 4, entry threshold of *P*_Uncorrected_<.001, K_E_=10.

| **Anatomic Region** | **Cluster Size (mm^3^)** | **Peak MNI Coordinates** | | | ***Z*** |
| --- | --- | --- | --- | --- | --- |
|  |  | **x** | **y** | **z** |  |
| Occipital pole, calcarine, lingual (bilateral) | 1405 | -10 | -100 | 4 | Inf |
| Middle temporal gyrus, sup temp, angular, inferior occ | 1744 | 56 | -46 | -4 | 6.59 |
| Middle temporal gyrus, sup temp | 1172 | -54 | -52 | -2 | 5.59 |
| vMPFC, gyrus rectus, frontal pole | 350 | -2 | 60 | -10 | 5.21 |
| Middle temporal gyrus, sup temp, temporal pole | 159 | -50 | 0 | -24 | 5.05 |
| Opercular part of the inferior frontal gyrus, triangular part, orbital part, lateral orbital gyrus, temporal pole, frontal operculum, posterior orbital gyrus | 669 | 58 | 20 | 6 | 5.02 |
| Inferior temporal gyrus | 81 | 40 | -8 | -50 | 4.65 |
| Temporal pole | 41 | 44 | 4 | -32 | 4.38 |
| Opercular part of the inferior frontal gyrus, triangular part, posterior orbital gyrus, lateral orbital gyrus, temporal pole | 572 | -52 | 18 | 2 | 4.26 |
| Supplementary motor cortex, dMPFC | 121 | 6 | 26 | 58 | 4.13 |
| Thalamus | 12 | -10 | -32 | 2 | 4.09 |
| Lateral orbital gyrus, orbital part of the inferior frontal gyrus | 27 | -48 | 32 | -18 | 3.90 |
| Postcentral gyrus | 33 | -50 | -18 | 52 | 3.90 |
| Superior frontal gyrus | 27 | -12 | 42 | 50 | 3.84 |
| Temporal pole | 38 | -36 | 26 | -34 | 3.82 |
| Inferior temporal gyrus | 62 | -48 | 0 | -48 | 3.81 |
| Orbital part of the inferior frontal gyrus, lateral orbital gyrus | 38 | 46 | 34 | -16 | 3.78 |
| Middle frontal gyrus | 41 | -46 | 10 | 46 | 3.71 |
| ACC | 10 | -12 | 38 | -10 | 3.59 |

*Supplementary Table 6.* Significant activation and deactivation associated with relatedness (relate very much>relate not at all contrast and relate very much>relate somewhat contrasts). Activation thresholded at *P*_FDR_<.05, K_E_=10.

| **Anatomic Region** | **Cluster Size (mm^3^)** | **Peak MNI Coordinates** | | | ***Z*** |
| --- | --- | --- | --- | --- | --- |
|  |  | **x** | **y** | **z** |  |
| ***Relate Very Much>Relate Not At All Activation*** |  |  |  |  |  |
| *Postcentral gyrus | 314 | -42 | -28 | 62 | 4.99 |
| vMPFC | 474 | -6 | 54 | -14 | 4.87 |
| Superior frontal gyrus | 275 | -24 | 32 | 52 | 4.66 |
| Occipital pole | 122 | 14 | -100 | -2 | 4.36 |
| Calcarine cortex | 56 | -12 | -98 | -2 | 4.31 |
| Precuneus | 80 | -4 | -54 | 18 | 4.29 |
| vMPFC | 19 | 2 | 38 | -18 | 4.15 |
| Ventral diencephalon | 14 | -6 | -14 | -16 | 3.95 |
| Cerebellum | 20 | 18 | -50 | -28 | 3.95 |
| Anterior insula | 15 | -36 | 8 | -10 | 3.82 |
| ***Relate Very Much>Relate Not At All Deactivation*** |  |  |  |  |  |
| *Postcentral gyrus | 106 | -54 | -18 | 54 | 6.11 |
| ***Relate Very Much>Relate Somewhat Activation*** |  |  |  |  |  |
| vMPFC | 1220 | -6 | 50 | 6 | 4.79 |
| Postcentral gyrus | 511 | -42 | -28 | 66 | 4.45 |
| Central operculum | 65 | -40 | -2 | 12 | 4.28 |
| Posterior orbital gyrus | 302 | -30 | 26 | -18 | 4.28 |
| Putamen | 100 | -30 | -10 | -10 | 4.23 |
| Angular gyrus | 106 | -42 | -62 | 24 | 4.06 |
| PCC | 230 | -10 | -42 | 28 | 3.98 |
| Superior frontal gyrus | 174 | -18 | 30 | 46 | 3.92 |
| Cerebellum | 30 | 22 | -50 | -26 | 3.92 |
| Putamen | 37 | -28 | -18 | 6 | 3.88 |
| Opercular part of the inferior frontal gyrus | 41 | -52 | 22 | 10 | 3.75 |
| Supramarginal gyrus | 31 | -54 | -50 | 20 | 3.68 |
| Caudate | 19 | -6 | 8 | 6 | 3.61 |
| Posterior orbital gyrus | 10 | 32 | 26 | -20 | 3.60 |
| Occipital pole | 18 | 10 | -94 | 6 | 3.58 |
| Thalamus | 10 | -18 | -18 | 14 | 3.56 |
| Thalamus | 10 | -12 | -14 | 0 | 3.53 |
| Ventral diencephalon | 21 | 2 | -10 | -10 | 3.53 |

*Clusters that also survive whole-brain FWE correction for multiple comparisons

*Supplementary Table 7.* Significant deactivation associated with eye distance ratings (eyes far apart>eyes close together contrast). Activation thresholded at *P*_FDR_<.05, K_E_=10.

| **Anatomic Region** | **Cluster Size (mm^3^)** | **Peak MNI Coordinates** | | | ***Z*** |
| --- | --- | --- | --- | --- | --- |
|  |  | **x** | **y** | **z** |  |
| *Middle frontal gyrus, superior frontal gyrus | 21 | 30 | 14 | 62 | 5.59 |

*Clusters that also survive whole-brain FWE correction for multiple comparisons


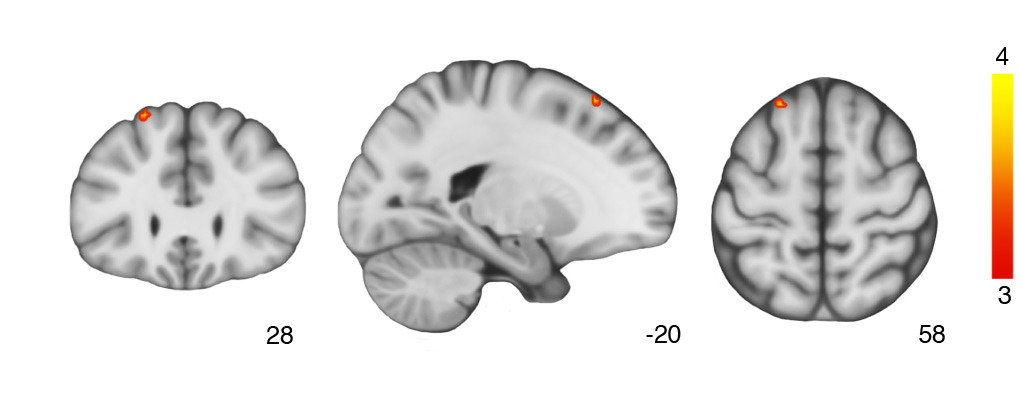


*Supplementary Figure 1.* Activation map representing the correlation between brain activity related to increasing relatedness and SCIM scores. Small volume correction applied, relate very much>relate not at all thresholded at *P_FDR_*<.05, K_E_=10, entry threshold *P_Uncorrected_*<.001. Left = left. Coordinates = -20, 28, 58.
